# Factorial Invariance of the Entrepreneurial Intentions Scale Among Colombian and Ecuadorian University Students

**DOI:** 10.3390/bs16071233

**Published:** 2026-07-20

**Authors:** Ignacio Norambuena-Paredes, José Luis Gálvez-Nieto, Eulalia Elizabeth Salas-Tenesaca, Cristina Tavera-Cuellar, Constanza Norambuena-Candia

**Affiliations:** 1 Departamento de Trabajo Social, Universidad de La Frontera, Temuco 4780000, Chile; ignacio.norambuena@ufrontera.cl; 2 Departamento de Ciencias Empresariales, Universidad Técnica Particular de Loja, Loja 110150, Ecuador; eesalas@utpl.edu.ec; 3 Facultad de Administración de Empresas, Universidad Santo Tomas Bucaramanga, Bucaramanga 681011, Colombia; cristina.tavera@ustabuca.edu.co; 4 Programa Magister en Educación Física y Vida Saludable, Universidad de La Frontera, Temuco 4780000, Chile; c.norambuena01@ufromail.cl

**Keywords:** entrepreneurship, entrepreneurial intention, entrepreneurial capability, factorial invariance, psychometric properties

## Abstract

Entrepreneurial intention is a key antecedent of entrepreneurial behavior and an essential construct for understanding the educational and occupational trajectories of university students. In comparative research, its assessment requires psychometrically equivalent instruments to ensure valid cross-national comparisons. This study aimed to evaluate the psychometric equivalence of the Entrepreneurial Intentions Scale among Colombian and Ecuadorian university students. A quantitative, instrumental, and cross-sectional design was employed with a sample of 803 students, including 319 Colombians and 484 Ecuadorians. Participants completed the six-item Entrepreneurial Intentions Scale using a seven-point Likert response format. Descriptive analyses, confirmatory factor analysis, configural, metric, and scalar invariance testing, latent mean comparisons, and reliability analyses using McDonald’s Omega and Cronbach’s Alpha were conducted. The results supported a unidimensional structure in both samples, with positive and statistically significant factor loadings. Invariance analyses demonstrated adequate model fit, confirming configural, metric, and scalar equivalence across countries. Latent mean comparisons revealed no statistically significant differences between Colombian and Ecuadorian students. Furthermore, the scale exhibited high internal consistency in both groups. These findings demonstrate that the Entrepreneurial Intentions Scale is a valid, reliable, and measurement-invariant instrument for cross-national research involving Latin American university students.

## 1. Introduction

Entrepreneurial intention is a key antecedent of new venture creation and represents one of the central constructs in entrepreneurship research (Du, 2024; Duan, 2022; Zhuang & Sun, 2025). This construct has attracted growing global interest due to its relevance in fostering economic development, innovation, and the active participation of young people within entrepreneurial ecosystems (Pérez et al., 2026; Zhang & Ji, 2024). However, despite the widespread use of instruments designed to assess entrepreneurial intention, empirical evidence regarding their factorial invariance remains limited, particularly among young populations. This issue is critical for ensuring the validity and comparability of findings in cross-cultural research (Cuesta et al., 2018; Liñán & Chen, 2009; Norambuena et al., 2025; Schelfhout et al., 2016; Setiawan & Basri, 2023).

Entrepreneurial intention is defined as an individual’s conscious and deliberate willingness to initiate an entrepreneurial activity, representing the most immediate antecedent of entrepreneurial behavior (Liguori et al., 2024; Soares & de Melo, 2024). From the perspective of the Theory of Planned Behavior, entrepreneurial intention is understood as the outcome of the interaction between attitudes toward entrepreneurship, subjective norms, and perceived behavioral control, factors that shape the cognitive evaluation of opportunities and risks associated with venture creation (Ajzen, 1991; Nayak et al., 2024; Norambuena et al., 2025).

In this regard, entrepreneurial intentions are shaped by beliefs, motivations, values, and self-efficacy perceptions, as well as by contextual influences such as social support, environmental conditions, and the availability of resources, all of which influence the decision to engage in entrepreneurial activity (Guevara-Otero et al., 2026; Wiyono & Wu, 2022). As highlighted in the scientific literature, entrepreneurial intention constitutes a fundamental component of entrepreneurial behavior, as it reflects the psychological commitment that precedes action and enables the prediction of the likelihood that individuals will transform their ideas into entrepreneurial initiatives (Mehta et al., 2024; Shetty et al., 2024).

From a developmental perspective, entrepreneurial intention acquires particular relevance during youth, a stage characterized by vocational decision-making processes, the construction of life projects, and the definition of career trajectories (Ahmad et al., 2025; Choi & McLain, 2024; Shirokova et al., 2022). During this period, the propensity toward entrepreneurship is influenced by educational experiences, future expectations, and the sociocultural context in which young people are embedded, which may lead to significant variations in how the construct is understood and expressed (Arango-Botero et al., 2022; Pérez-Álvarez et al., 2022; Renart-Vicens et al., 2022). Consequently, the assessment of entrepreneurial intention among young populations requires instruments capable of capturing the meaning of the construct equivalently across different groups and cultural contexts (Laguía et al., 2017; Norambuena et al., 2025).

Within this framework, the rigorous assessment of entrepreneurial intention among young populations poses important challenges for behavioral science research (Muñiz Fernández et al., 2014; Norambuena et al., 2025), particularly when comparisons are made across different cultural or sociodemographic groups (Laguía et al., 2017; Rueda et al., 2015). The lack of robust empirical evidence regarding the equivalence of measurement instruments may compromise the interpretation of findings and limit the generalizability of results. Therefore, further psychometric analyses are needed to ensure that the scales employed assess the construct consistently and comparably across groups. Such efforts contribute to a more accurate understanding of entrepreneurial intention and provide robust evidence to inform the development of policies and interventions based on reliable data (Norambuena et al., 2025).

In the Latin American context, the study of entrepreneurial intention among university students is particularly relevant due to the structural similarities, as well as the pronounced socioeconomic, educational, and institutional differences, between countries such as Colombia and Ecuador (Laguía et al., 2017; Lopez et al., 2021; Novelo et al., 2021; Soares & de Melo, 2024). These nations share challenges related to youth unemployment, labor market informality, and the need to diversify their economies, which has positioned entrepreneurship as a key strategy for economic and social development (Alicia et al., 2025; Albiter et al., 2024; Karali et al., 2023).

Despite the widespread use of the Entrepreneurial Intentions Scale in international research, there is still limited evidence regarding whether the instrument measures entrepreneurial intention equivalently across Latin American countries. Without evidence of measurement invariance, comparisons between countries may reflect measurement bias rather than true differences in entrepreneurial intention. Therefore, establishing the psychometric equivalence of the scale constitutes a fundamental prerequisite for valid cross-national comparisons.

Within this context, examining entrepreneurial intention among university students in Colombia and Ecuador provides an opportunity to deepen understanding of how this construct manifests across Latin American settings characterized by diverse historical, cultural, and socioeconomic trajectories, yet shaped by common challenges (Hinojosa, 2026; Ojeda-Beltrán et al., 2023; Pinos-Ramón et al., 2024). Exploring these realities helps highlight the role of entrepreneurship as a meaningful pathway for personal development, social inclusion, and economic participation among young people, while also advancing knowledge of the psychosocial factors that shape the propensity to engage in entrepreneurial activities (Alicia et al., 2025; Albiter et al., 2024; Karali et al., 2023). In this regard, the present study contributes to the field of behavioral sciences by providing evidence that supports a more contextualized and comparative understanding of entrepreneurial intention, thereby strengthening academic dialogue and informing the design of university policies and entrepreneurship education programs.

### Entrepreneurial Intentions Scale in Young People: Description and Theoretical Structure of the Construct

The Entrepreneurial Intentions Scale was originally developed by Liñán and Chen (2009) with the aim of providing a standardized, theoretically grounded, and psychometrically robust instrument for assessing entrepreneurial intention among university students (Norambuena et al., 2025). Its development addressed previous limitations in the measurement of the construct, which were characterized by the use of ad hoc instruments and limited comparability across studies. Consequently, the scale has become a valid tool for the systematic assessment of entrepreneurial intention across diverse cultural contexts (Blanco-Mesa et al., 2024; Galleguillos-Cortés et al., 2019; Lechuga-Nevárez & Vázquez-Rueda, 2021).

This scale emerged within the framework of theoretical and methodological efforts aimed at understanding entrepreneurship as a psychological process that precedes entrepreneurial action, particularly among young people and university students (Li & Chen, 2022; Xu & An, 2024; Yin et al., 2022). Its development was driven by the need to operationalize entrepreneurial intention as a latent construct, conceptualized as the immediate cognitive antecedent of entrepreneurial behavior (Huang et al., 2024; Norambuena et al., 2025; Wang et al., 2022). In this regard, the scale is grounded in a research tradition that views the decision to engage in entrepreneurship not as a spontaneous event, but rather as the outcome of evaluative, motivational, and contextual processes that can be systematically assessed through psychometrically robust instruments (Guevara-Otero et al., 2026; Wiyono & Wu, 2022).

From a theoretical perspective, the scale is primarily grounded in the Theory of Planned Behavior, which has demonstrated strong explanatory power in predicting intentional behaviors across various social domains (Pan et al., 2026; Yeo et al., 2024). Within the field of entrepreneurship, this theory posits that the intention to start a business is shaped by attitudes toward entrepreneurial behavior, subjective norms, and perceived behavioral control (Mitra et al., 2023; Zwane & Osuigwe, 2024). These dimensions encompass personal evaluations of entrepreneurship, perceptions of social support or pressure, and beliefs regarding one’s own ability to engage in entrepreneurial activities, forming a theoretical model that has been extensively validated in studies involving university students and young adults (Akram & Hye, 2026; Frese & Gielnik, 2023).

Although the Entrepreneurial Intentions Scale was developed based on the Theory of Planned Behavior, its usefulness extends beyond assessing entrepreneurial intention as an isolated construct. From this perspective, the scale represents an operational measure of the intentional component proposed by Ajzen (1991), enabling the assessment of the cognitive disposition that precedes entrepreneurial behavior. Consequently, the validity of inferences drawn from its scores depends on the instrument’s ability to consistently represent this theoretical construct across the different contexts in which it is applied.

Accumulated empirical evidence consistently supports the validity of the Entrepreneurial Intentions Scale across diverse educational and cultural contexts (Barba-Sánchez et al., 2022; Do-Nguyen & Nguyen, 2023; Ridwan et al., 2025). Previous studies have confirmed a stable factorial structure, along with adequate model fit, reliability, and convergent validity indices (Liñán & Chen, 2009; Norambuena et al., 2025). Furthermore, findings have demonstrated significant associations between entrepreneurial intention and relevant variables such as entrepreneurship education, self-efficacy, future expectations, and perceived opportunities, reinforcing the scale’s relevance as a measurement tool in both applied and comparative research (Branca et al., 2025; Guevara-Gómez et al., 2022; Martins et al., 2023).

Despite its widespread use and strong empirical support, there remains a need for further examination of the scale’s psychometric equivalence across different sociocultural contexts. The absence of measurement invariance studies may limit the comparative interpretation of findings, as observed differences between groups could reflect measurement bias rather than genuine variations in the underlying construct (Ferreira-Neto et al., 2023; Ghouse et al., 2024; Otache et al., 2024). In this context, factorial invariance analyses are of particular importance in cross-cultural research, especially in regions such as Latin America, which are characterized by substantial social, educational, and economic heterogeneity.

From a methodological perspective, comparisons of entrepreneurial intentions across countries are only valid when there is evidence that the instrument measures the same construct equivalently across groups. The absence of measurement equivalence may lead to misleading interpretations, whereby observed differences are attributed to cultural or contextual factors when they may actually result from differential item functioning. Therefore, establishing measurement equivalence constitutes a fundamental prerequisite for conducting cross-cultural research and for informing evidence-based entrepreneurship education policies grounded in valid cross-national comparisons.

Despite the widespread use of the Entrepreneurial Intentions Scale across diverse cultural contexts, the available evidence has primarily focused on confirming its factorial structure and internal consistency within single-country samples. Consequently, evidence regarding the measurement equivalence of the instrument across Latin American countries remains limited, despite this being a fundamental methodological requirement for conducting valid cross-cultural comparisons. In the absence of evidence supporting measurement invariance, observed differences between groups may reflect measurement bias rather than genuine differences in entrepreneurial intentions, thereby compromising the validity of conclusions drawn from comparative studies. In this context, the aim of the present study was to evaluate the psychometric properties and measurement invariance of the Entrepreneurial Intentions Scale among university students in Colombia and Ecuador. By providing evidence of the instrument’s measurement equivalence across these two national contexts, this study strengthens the methodological foundation for cross-cultural research in Latin America, enabling more rigorous comparisons of entrepreneurial intentions and supporting the development of comparative research and evidence-based entrepreneurship education policies.

## 2. Materials and Methods

### 2.1. Participants

The study included 803 university students from seven public and private universities in Colombia and Ecuador, selected using a non-probability convenience sampling approach. Institutional contacts at the participating universities agreed to collaborate in the study and distributed an invitation containing a link to the online questionnaire hosted on the QuestionPro platform. Participation was voluntary and anonymous. Data were collected through the QuestionPro platform, which was configured to accept only fully completed questionnaires, thereby ensuring that no missing data were present in the analysed dataset. As participation was voluntary and based on convenience sampling, it was not possible to determine the response rate accurately, and the sample was not intended to be statistically representative of the university student population in Colombia and Ecuador.

The Colombian sample consisted of 319 university students (36.4% men and 63.6% women), aged between 18 and 29 years (M = 21.76; SD = 2.40). Participants were enrolled in four public and private universities located in the cities of Bogotá and Bucaramanga. The Ecuadorian sample comprised 484 university students (50.8% men and 49.2% women), aged between 18 and 29 years (M = 21.64; SD = 2.57). Participants were recruited from three public and private universities located in the city of Quito.

To examine the equivalence of the two samples, differences in sex distribution were assessed using the chi-square test, whereas differences in age were analysed using the independent-samples *t*-test. The results showed no statistically significant difference in mean age between students from the two countries (t = 0.85, *p* = 0.396). Although the chi-square test indicated a statistically significant difference in sex distribution (χ^2^(1) = 16.25, *p* < 0.001), the associated effect size was small (Cramér’s V = 0.142), indicating only minor demographic differences between the two samples. Given the relatively large sample size, this finding is likely to reflect the high statistical power of the test rather than a substantively meaningful difference. Therefore, the samples were considered sufficiently comparable for the subsequent cross-national psychometric analyses.

The sample size was considered adequate for confirmatory factor analysis, measurement invariance testing, and reliability estimation, providing stable parameter estimates and sufficient statistical power for the planned psychometric analyses.

### 2.2. Instrument

To address the objectives of the study, a sociodemographic questionnaire was administered to characterize the study sample. This instrument collected relevant information on variables such as age, sex, socioeconomic status, country of origin, and other pertinent contextual characteristics, which are essential for providing an adequate description of the participants and for the subsequent interpretation of the empirical findings.

Entrepreneurial intention was assessed using the Entrepreneurial Intentions Scale developed by Liñán and Chen (2009) (see Supplementary Materials), a widely used instrument in international research on entrepreneurial behavior. The scale evaluates entrepreneurial intention through six items that assess individuals’ perceptions regarding entrepreneurship and their personal willingness to engage in entrepreneurial activities. Responses are recorded on a seven-point Likert scale ranging from 1 (strongly disagree) to 7 (strongly agree), allowing for the assessment of varying degrees of endorsement of the attitudes under examination.

The scale has demonstrated strong psychometric properties across different Latin American contexts. Previous studies have reported adequate internal consistency, with a Cronbach’s Alpha coefficient of 0.91 in a Colombian sample (Laguía et al., 2017) and a McDonald’s Omega coefficient of 0.94 in a Mexican population (Norambuena et al., 2025). These findings support the reliability of the instrument and confirm its suitability for assessing entrepreneurial intention in the present study.

### 2.3. Procedure

For the administration of the Entrepreneurial Intentions Scale, formal contact was established with the directors of the participating universities, who granted the necessary authorization for the implementation of the study. The research received prior approval from the Ethics Committee of the University of La Frontera (UFRO File No. 145/23), ensuring compliance with ethical principles governing research involving human participants.

Data were collected through an online questionnaire hosted on the QuestionPro platform. Participants were invited to take part in the study via email invitations sent by the research team, which included a link to the questionnaire and the informed consent form. This document outlined the objectives of the study, the voluntary nature of participation, the confidentiality and anonymity of the information provided, the absence of any associated risks, and participants’ right to withdraw from the study at any time without any consequences.

Additionally, to ensure the linguistic and cultural appropriateness of the instrument, five expert judges from Colombia and Ecuador evaluated the items of the Entrepreneurial Intentions Scale, reviewing the potential presence of colloquial expressions or semantic ambiguities. Following this content validation process, the judges concluded that the scale was clear, comprehensible, and appropriate for use in both national contexts. Consequently, no modifications were required, and the items were retained in their original form for both countries. Data collection was conducted during September 2025.

### 2.4. Data Analysis

First, descriptive analyses were conducted for each item of the Entrepreneurial Intentions Scale, including the mean and standard deviation for the Colombian, Ecuadorian, and total samples. Additionally, item normality was examined using the Kolmogorov–Smirnov test. Subsequently, a confirmatory factor analysis (CFA) was performed in each sample based on the instrument’s previously established theoretical structure, with the aim of evaluating the validity of its factorial structure. The analyses were conducted using Mplus version 7.11 (Muthén & Muthén, 1998–2012, Los Angeles, CA, USA), employing the robust maximum likelihood (MLR) estimation method.

Because the data did not meet the assumption of multivariate normality, the use of the robust maximum likelihood (MLR) estimator was deemed appropriate for obtaining robust parameter estimates and model fit indices. This decision was supported by the results of Mardia’s test, which indicated deviations from multivariate normality in both the Colombian (1.10) and Ecuadorian (1.14) samples (Mardia, 1970). In this context, the use of robust maximum likelihood estimation enables the generation of reliable fit indices and accurate estimates of parameters and standard errors (Finney & DiStefano, 2006; Flora & Curran, 2004).

To evaluate model fit, several goodness-of-fit indices were considered: the Satorra–Bentler chi-square (SB-χ^2^; Satorra & Bentler, 2001), the Comparative Fit Index (CFI), the Tucker–Lewis Index (TLI), and the Root Mean Square Error of Approximation (RMSEA). The criteria for acceptable model fit were CFI and TLI values greater than 0.90 and RMSEA values below 0.08, which are considered indicative of a reasonable model fit (Schumacker & Lomax, 2004; Browne & Cudeck, 1992).

Subsequently, the level of factorial invariance of the scale was examined across the two country samples using a hierarchical sequence of measurement invariance models (Vandenberg & Lance, 2000). Specifically, configural invariance was assessed to determine equivalence in the number of factors and item structure; metric invariance was evaluated to test the equality of factor loadings; scalar invariance was examined to verify the equivalence of item intercepts; and latent mean invariance was assessed to compare latent factor means across groups. Measurement invariance was evaluated according to the criteria proposed by Chen (2007), considering ΔTLI = 0 as indicative of perfect fit and ΔTLI ≤ 0.01 as evidence of acceptable fit, as well as ΔRMSEA ≤ 0.015 as evidence supporting measurement invariance.

Finally, to ensure the internal consistency of the instrument, factor reliability was assessed using McDonald’s Omega coefficient and standardized Cronbach’s Alpha (Elosua & Zumbo, 2008), together with corrected item–total correlations. This approach provided a comprehensive evaluation of the scale’s reliability and internal consistency.

## 3. Results

### 3.1. Descriptive and Correlational Analyses

Table 1 presents the descriptive statistics for the entrepreneurial intention items in Colombia and Ecuador. Using a response scale ranging from 1 to 7, both samples exhibited high mean scores across all items, indicating elevated levels of entrepreneurial intention in both countries. Differences between Colombia and Ecuador were minimal and showed no systematic pattern, with slightly higher scores observed in Ecuador for some items (e.g., It1 and It2) and marginally higher scores in Colombia for others (e.g., It4 and It6).

In both groups, skewness values were consistently negative, suggesting a concentration of responses toward the upper end of the scale. Meanwhile, kurtosis values remained within acceptable ranges, whereas the Kolmogorov–Smirnov test indicated statistically significant deviations from normality for all items. Given the relatively large sample size, these results were interpreted with caution, and the assessment of skewness and kurtosis suggested no substantial departures from univariate normality. Standard deviations were comparable across countries, indicating a similar degree of variability in participants’ responses.

Inter-item correlations were positive and statistically significant in both countries (Table 2). Correlation coefficients ranged from moderate to high in both samples, with slightly stronger associations observed in the Ecuadorian sample. Overall, the correlation pattern was consistent across groups, providing support for the internal coherence of the entrepreneurial intention construct.

### 3.2. Factorial Structure of the Entrepreneurial Intentions Scale

Confirmatory factor analyses were conducted separately for the Colombian and Ecuadorian samples. In both groups, all factor loadings were positive, statistically significant (*p* < 0.001), and of moderate to high magnitude, providing support for the unidimensional structure of the scale. As shown in Table 3, the standardized factor loadings were consistently high across both national samples. Regarding model fit, both samples demonstrated adequate to excellent fit. The Colombian sample showed excellent incremental fit indices, although the RMSEA indicated a marginal fit. In contrast, the Ecuadorian sample exhibited excellent fit across all indices. Overall, these findings provide support for the factorial validity of the Entrepreneurial Intentions Scale in both countries.

### 3.3. Factorial Invariance

The results presented in Table 4 support the measurement invariance of the Entrepreneurial Intentions Scale across Colombia and Ecuador. Both configural and metric invariance models demonstrated excellent fit, indicating equivalent factorial structures and factor loadings across countries. Scalar invariance was also confirmed, as changes in the CFI remained within the recommended thresholds, despite a slight increase in RMSEA. Overall, these findings suggest that the scale operates equivalently across groups, thereby allowing meaningful comparisons of latent means between countries.

Although the scalar model showed an increase in RMSEA relative to the metric model, the absolute RMSEA value remained within the range of good model fit, while the CFI, TLI, and SRMR continued to indicate excellent model fit. Furthermore, the change in CFI remained well below the criterion proposed by Chen (2007), supporting the acceptance of scalar invariance.

Latent mean differences in entrepreneurial intention were examined after scalar invariance had been established between Colombia and Ecuador. The latent mean of the reference group (Colombia) was fixed at zero, whereas the latent mean of the Ecuadorian group was freely estimated. The results revealed no statistically significant differences between the two countries (β = −0.018, z = −0.233, *p* = 0.816, 95% CI [−0.167, 0.132]). Moreover, the estimated latent mean difference was negligible, indicating a trivial magnitude of difference between the two groups and supporting comparable levels of entrepreneurial intention across both samples.

### 3.4. Reliability Evidence

Reliability analyses indicated high internal consistency of the Entrepreneurial Intentions Scale in both countries. In the Colombian sample, McDonald’s Omega and Cronbach’s Alpha were 0.940 and 0.938, respectively, whereas both coefficients reached 0.937 in the Ecuadorian sample. Confidence intervals were narrow and showed substantial overlap across groups, suggesting stable and comparable reliability estimates. Overall, these findings provide strong evidence for the internal consistency of the scale in both samples are presented in Table 5.

## 4. Discussion

The findings of this study provide robust psychometric evidence regarding the equivalence of the Entrepreneurial Intentions Scale among university students in Colombia and Ecuador. Overall, the results support the proposed hypothesis by confirming a stable unidimensional structure, high and statistically significant factor loadings, adequate model fit indices, and satisfactory levels of factorial invariance across both countries. This evidence is particularly relevant in the field of cross-cultural research, where score comparability cannot be assumed solely on the basis of translating or administering an instrument but must be empirically demonstrated through measurement invariance analyses (Vandenberg & Lance, 2000; Chen, 2007). From this perspective, the present study contributes to strengthening the validity of the instrument developed by Liñán and Chen (2009), extending its applicability to Latin American contexts characterized by heterogeneous educational, economic, and institutional trajectories.

The confirmation of the scale’s unidimensional structure across both samples represents a central finding, as it indicates that the six items converge on a common factor reflecting a conscious and deliberate disposition toward entrepreneurship. This result is consistent with the instrument’s original conceptualization, which was designed to capture entrepreneurial intention as a psychological construct that precedes entrepreneurial action (Liñán & Chen, 2009), and with recent studies reporting a stable factorial structure among Latin American university students (Laguía et al., 2017; Norambuena et al., 2025). Furthermore, the moderate to high factor loadings observed in both Colombia and Ecuador suggest that the items adequately represent the underlying latent construct, reinforcing the theoretical relevance of the model and its alignment with the Theory of Planned Behavior, according to which intention constitutes the immediate antecedent of entrepreneurial behavior (Ajzen, 1991; Duan, 2022; Nayak et al., 2024).

Descriptive results revealed high levels of entrepreneurial intention in both samples, with only minimal and non-systematic differences between countries. However, because the present study focused on evaluating the psychometric properties and measurement invariance of the scale, these findings should not be interpreted as evidence regarding the underlying determinants of entrepreneurial intention. Rather, they indicate that the instrument provides comparable measurements across both national contexts, allowing valid cross-country comparisons in future explanatory studies. Recent literature has highlighted that entrepreneurial intention among university students in Latin America is shaped by the intersection of personal aspirations, academic training, perceived opportunities, and structural constraints within the labor market (Lopez et al., 2021; Soares & de Melo, 2024). In this regard, the similarity in scores should not be interpreted as evidence of complete contextual homogeneity but rather as an indication of a comparable psychological pattern regarding the propensity to engage in entrepreneurial activities.

Evidence of configural invariance indicates that the Entrepreneurial Intentions Scale retains the same factorial structure across Colombian and Ecuadorian university students. This finding suggests that entrepreneurial intention is conceptualized similarly in both groups and provides the methodological foundation for subsequent tests of measurement invariance. Consequently, the scale can be meaningfully evaluated across these national contexts within cross-cultural psychometric research (Vandenberg & Lance, 2000). Consequently, these results strengthen the utility of the scale for comparative regional research and address a need highlighted in the literature for psychometrically sound instruments capable of assessing entrepreneurial competencies, attitudes, and intentions among young populations (Cuesta et al., 2018; Schelfhout et al., 2016; Setiawan & Basri, 2023).

Metric invariance further demonstrates that the factor loadings are equivalent across countries, indicating that each item contributes similarly to the measurement of entrepreneurial intention in both samples. This finding supports valid comparisons of associations between entrepreneurial intention and other variables in future cross-cultural studies, reducing the likelihood that observed differences are attributable to measurement artifacts. This indicates that each item contributes to the measurement of the latent factor to a similar extent in both countries, allowing associations between entrepreneurial intention and other psychological, educational, or contextual variables to be compared without being distorted by differences in item functioning. This finding creates opportunities for more complex explanatory studies in the region, particularly those aimed at examining the role of entrepreneurship education, self-efficacy, motivation, social support, and perceived opportunities in the development of entrepreneurial intention (Barba-Sánchez et al., 2022; Do-Nguyen & Nguyen, 2023; Martins et al., 2023). Therefore, the scale is not only useful for describing levels of entrepreneurial intention but also for testing comparative theoretical models with appropriate psychometric guarantees.

The establishment of scalar invariance enabled valid comparisons of latent means between countries. Consistent with this level of measurement equivalence, no statistically significant latent mean differences were observed between the Colombian and Ecuadorian samples. These findings indicate comparable entrepreneurial intention scores within the study population and support the use of the scale for future comparative research across similar contexts. This level of equivalence is essential for comparing latent means because it ensures that any observed differences—or lack thereof—between countries reflect genuine variations in the construct rather than systematic measurement bias (Chen, 2007). In the present study, latent mean comparisons revealed no statistically significant differences between Colombia and Ecuador, suggesting comparable levels of entrepreneurial intention among university students in both countries. This finding is particularly noteworthy because it indicates that, after establishing scalar invariance, latent mean comparisons indicated no statistically significant differences between the Colombian and Ecuadorian samples. These findings suggest comparable latent scores within the studied samples. However, they should not be interpreted as evidence that entrepreneurial intention develops similarly across broader national contexts, since the present study was designed to evaluate measurement equivalence rather than explanatory mechanisms. This result is consistent with previous research highlighting the cross-cutting role of entrepreneurship as an educational and occupational aspiration among university students and young adults (Choi & McLain, 2024; Shirokova et al., 2022; Zhuang & Sun, 2025).

Regarding reliability, both McDonald’s Omega and Cronbach’s Alpha coefficients indicated high internal consistency across countries, with very similar estimates obtained in the Colombian and Ecuadorian samples. This finding reinforces the internal stability of the instrument and confirms that the items demonstrate adequate coherence in assessing entrepreneurial intention as a unidimensional construct. The joint use of Omega and Alpha represents a methodological strength, as it provides a more comprehensive assessment of internal consistency and avoids relying exclusively on Cronbach’s Alpha, whose interpretation may be affected by the restrictive assumption of tau-equivalence (Elosua & Zumbo, 2008). From a comparative perspective, these results are consistent with previous findings from Colombia and Mexico, where the scale has also demonstrated high reliability coefficients (Laguía et al., 2017; Norambuena et al., 2025), thereby strengthening the regional empirical evidence supporting its use in university-based research.

Taken together, these findings support the Entrepreneurial Intentions Scale as a valid, reliable, and measurement-invariant instrument for comparative research involving university students in Colombia and Ecuador. The evidence provided by this study establishes a robust methodological basis for future psychometric and explanatory research conducted in comparable university populations. Although the present findings may contribute to advancing research on youth entrepreneurship in Latin America, their broader regional application should be supported by additional evidence from other countries and cultural settings (Frese & Gielnik, 2023; Liguori et al., 2024; Shirokova et al., 2022).

## 5. Limitations and Future Research

A first limitation of the present study is its cross-sectional design, which allows for the assessment of the factorial structure, reliability, and measurement equivalence of the Entrepreneurial Intentions Scale at a specific point in time but does not permit the examination of the construct’s temporal stability or changes in entrepreneurial intention over time. This limitation is particularly relevant given that entrepreneurial intention is not a static disposition but rather a psychological process that may evolve as a function of educational experiences, exposure to entrepreneurship programs, changes in self-efficacy, perceived opportunities, and institutional conditions (Ajzen, 1991; Frese & Gielnik, 2023; Shirokova et al., 2022). In this regard, future research should incorporate longitudinal designs to examine the test–retest stability of the instrument, as well as the development of entrepreneurial intention throughout the university trajectory, particularly before and after entrepreneurship-related educational interventions.

A second limitation concerns the generalizability of the findings. The sample consisted exclusively of university students from Colombia and Ecuador, which is consistent with the objectives of this psychometric study. Consequently, the results should not be generalized to other age groups, educational levels, academic disciplines, or non-university populations without additional empirical evidence. Although the present findings support the measurement equivalence of the Entrepreneurial Intentions Scale in these two national contexts, further validation studies are required before extending these conclusions to other populations (Laguía et al., 2017; Lopez et al., 2021; Soares & de Melo, 2024). Therefore, future studies should extend the validation of the instrument to more diverse samples, including students from both public and private institutions, non-university youth, recent graduates, and individuals who are already engaged in entrepreneurial activities.

A third limitation concerns the geographical generalizability of the findings. Although Colombia and Ecuador provide relevant contexts for evaluating the measurement equivalence of the scale, Latin America is characterized by considerable cultural, educational, economic, and institutional diversity. Therefore, the present findings should not be interpreted as evidence that the scale functions equivalently throughout the region. Additional measurement invariance studies across other Latin American countries are necessary before broader regional generalizations can be made (Chen, 2007; Vandenberg & Lance, 2000). Consequently, future research should extend factorial invariance analyses to other Latin American countries, including contexts with varying levels of economic development, university entrepreneurship policies, and youth labor market conditions, in order to advance toward a broader regional validation of the instrument.

A fourth limitation concerns the predominantly psychometric focus of the study. Although confirmatory factor analyses, measurement invariance testing, and reliability coefficients provide strong evidence regarding the technical quality of the instrument, these analyses do not, by themselves, explain the psychological, educational, and institutional mechanisms that shape entrepreneurial intention. According to the Theory of Planned Behavior, entrepreneurial intention is linked to attitudes, subjective norms, and perceived behavioral control, dimensions that may be influenced by factors such as entrepreneurial self-efficacy, social support, entrepreneurship education, motivation, perceived opportunities, and personal competencies (Ajzen, 1991; Barba-Sánchez et al., 2022; Do-Nguyen & Nguyen, 2023; Martins et al., 2023). Therefore, future research should integrate the scale into more complex explanatory models, incorporating mediating and moderating variables that facilitate a deeper understanding not only of the measurement of the construct but also of its antecedents and consequences.

As a future research direction, additional sources of validity evidence, such as convergent, discriminant, and criterion-related validity, should be examined to further strengthen the psychometric evaluation of the Entrepreneurial Intentions Scale. Incorporating these analyses would provide a more comprehensive assessment of the construct’s validity and reinforce confidence in its application across diverse educational and cultural contexts.

Finally, a fifth limitation relates to the use of self-report measures, which may be susceptible to social desirability bias, response styles, or the overestimation of individuals’ entrepreneurial disposition. This issue is particularly relevant in university settings, where entrepreneurship is often promoted as a desirable, innovative, and socially valued competency. Although self-report instruments are widely used in entrepreneurial intention research and have demonstrated adequate psychometric properties across different cultural contexts (Liñán & Chen, 2009; Laguía et al., 2017; Norambuena et al., 2025), future studies could complement quantitative assessments with mixed-methods approaches, interviews, focus groups, or behavioral indicators. Such strategies would facilitate the examination of the relationship between declared entrepreneurial intention and actual entrepreneurial behavior, recognizing that the transition from intention to action also depends on institutional conditions, available resources, and real opportunities for implementation (Liguori et al., 2024; Shirokova et al., 2022).

## 6. Conclusions

The findings of the present study indicate that the Entrepreneurial Intentions Scale demonstrates strong psychometric evidence supporting its use among university students in Colombia and Ecuador. The proposed unidimensional structure showed an adequate fit in both samples, with positive, statistically significant, and high factor loadings, confirming that the items consistently converge in measuring a single latent construct. These results support the theoretical and empirical robustness of the instrument and reinforce its suitability as a valid measure of entrepreneurial intention among university students in both countries.

Furthermore, the confirmation of configural, metric, and scalar invariance represents one of the main contributions of this study, demonstrating that the scale functions equivalently across both countries. This indicates that Colombian and Ecuadorian students interpret the construct of entrepreneurial intention through a common factorial structure, that the items contribute equivalently to the measurement of the latent factor, and that scores can be validly compared between the two groups. Consequently, the instrument provides methodological support for comparative studies between Colombia and Ecuador and offers evidence of psychometric equivalence for cross-cultural research conducted in these contexts.

The absence of statistically significant differences in latent means indicates comparable levels of entrepreneurial intention among the university students included in this study. These findings should be interpreted within the scope of the present psychometric evaluation and support the validity of comparisons between the two countries using the Entrepreneurial Intentions Scale. Overall, the results demonstrate that entrepreneurial intention exhibits an equivalent measurement pattern among university students in Colombia and Ecuador, facilitating future comparative research in these populations.

Finally, this study contributes to the advancement of psychometric research on entrepreneurial intention by providing evidence of factorial validity, reliability, and measurement invariance among university students in Colombia and Ecuador. From a practical perspective, the Entrepreneurial Intentions Scale can be used by educators to monitor students’ entrepreneurial intentions throughout their academic training, by universities to evaluate and strengthen entrepreneurship education initiatives, and by policymakers to assess the impact of entrepreneurship promotion programs in both countries. The demonstrated measurement equivalence also enables valid comparisons across institutional and national contexts in Colombia and Ecuador, providing a solid methodological foundation for future comparative research.

## Figures and Tables

**Table 1:** Descriptive statistics of entrepreneurial intention items by country.

Items	Mean	SD	Skewness	Kurtosis	K-S Test
Colombian Sample (*n* = 319)					
It1	4.72	1.67	−0.41	−0.37	0.148 **
It2	5.06	1.61	−0.60	−0.03	0.149 **
It3	5.28	1.53	−0.77	0.34	0.162 **
It4	5.38	1.56	−0.90	0.53	0.181 **
It5	5.22	1.61	−0.78	0.53	0.163 **
It6	5.38	1.51	−0.94	0.76	0.178 **
Ecuadorian Sample (*n* = 484)					
It1	4.90	1.70	−0.70	0.01	0.188 **
It2	5.10	1.57	−0.77	0.35	0.185 **
It3	5.28	1.56	−0.96	0.81	0.195 **
It4	5.29	1.55	−0.92	0.74	0.175 **
It5	5.16	1.51	−0.74	0.55	0.166 **
It6	5.28	1.51	−0.86	0.82	0.181 **

Note: K–S = Kolmogorov–Smirnov test. All Kolmogorov–Smirnov tests were statistically significant (*p* < 0.01). Given the relatively large sample size, these results were interpreted together with skewness and kurtosis to assess the univariate distribution of the items. And ** indicates statistical significance at the 0.01 level (*p* < 0.01) in the Kolmogorov–Smirnov test.

**Table 2:** Correlation matrix of entrepreneurial intention items by country.

Items	It1	It2	It3	It4	It5	It6
**It1**	—	0.586	0.576	0.588	0.551	0.516
**It2**	0.689	—	0.754	0.766	0.769	0.685
**It3**	0.571	0.740	—	0.867	0.848	0.774
**It4**	0.571	0.731	0.855	—	0.869	0.836
**It5**	0.577	0.730	0.783	0.839	—	0.814
**It6**	0.530	0.676	0.813	0.856	0.830	—

Note: Values above the diagonal correspond to the Colombian sample (*n* = 319), and values below the diagonal correspond to the Ecuadorian sample (*n* = 484). All correlations are statistically significant at *p* < 0.01.

**Table 3:** Confirmatory factor analysis (CFA) results for entrepreneurial intention by country.

Item	Colombia λ	Ecuador λ
It1	0.673	0.694
It2	0.85	0.854
It3	0.917	0.911
It4	0.985	0.941
It5	0.937	0.913
It6	0.932	0.909
Index	Colombia	Ecuador
Chi-square (df)	25.498 (9)	15.905 (9)
CFI	1	0.998
TLI	0.999	0.997
RMSEA	0.076	0.04
RMSEA CI 90%	[0.042–0.111]	[0.000–0.071]
SRMR	0.025	0.04

Note: λ = standardized factor loadings. SE = standard error. All factor loadings were statistically significant. CFI = Comparative Fit Index; TLI = Tucker–Lewis Index; RMSEA = Root Mean Square Error of Approximation; SRMR = Standardized Root Mean Square Residual.

**Table 4:** Measurement invariance of the entrepreneurial intention scale across Colombia and Ecuador (ULS estimation).

Modelo	χ^2^ (gl)	RMSEA	CFI	TLI	SRMR	ΔRMSEA	ΔCFI	Decisión
Configural	19.271 (18)	0.013	1.000	1.000	0.033	—	—	Aceptado
Métrico	20.580 (23)	0.000	1.000	1.000	0.034	−0.013	0.000	Aceptado
Escalar	93.192 (52)	0.044	0.994	0.997	0.036	0.044	−0.006	Aceptado

Note: ULS = unweighted least squares; χ^2^ = chi-square; gl = degrees of freedom; RMSEA = root mean square error of approximation; CFI = comparative fit index; TLI = Tucker–Lewis index; SRMR = standardized root mean square residual. ΔRMSEA and ΔCFI correspond to changes between nested models. Following conventional criteria (e.g., ΔCFI ≤ 0.01), configural, metric, and scalar invariance were supported across countries. The scalar model showed a slight increase in RMSEA; however, overall model fit remained acceptable.

**Table 5:** Reliability estimates of the entrepreneurial intention scale by country.

Country	McDonald’s ω	95% CI	Cronbach’s α	95% CI
Colombia	0.940	[0.929–0.950]	0.938	[0.926–0.948]
Ecuador	0.937	[0.928–0.946]	0.937	[0.928–0.946]

Note: ω = McDonald’s omega; α = Cronbach’s alpha; CI = confidence interval. All reliability estimates indicate high internal consistency across countries.

## Data Availability

The dataset for the study is available from the corresponding author upon reasonable request due to ethical restrictions.

## Supplementary Materials

The following supporting information can be downloaded at: https://www.mdpi.com/article/10.3390/bs16071233/s1, Escala Intención Emprendedora, utilizada en el estudio actual/Entrepreneurial Intentions Scale Used in the Present Study.
