# Supplementary material for: Factorial Invariance of the Entrepreneurial Intentions Scale Among Colombian and Ecuadorian University Students
Source: Behav Sci (Basel). 2026 Jul 20;16(7):1233. doi: 10.3390/bs16071233 (PMC13405597; doi:10.3390/bs16071233)
Supplement: Supplementary file 1 [file behavsci-16-01233-s001.zip › behavsci-4382958-supplementary.pdf]

Material suplementario/ Supplementary Material

Escala Intención Emprendedora, utilizada en el estudio actual / Entrepreneurial Intentions Scale Used in the Present Study

Instrucciones/ **Instructions:**

A continuación, encontrarás siete afirmaciones con las que puedes estar de acuerdo o en desacuerdo. Indica tu grado de acuerdo con cada afirmación / Below, you will find seven statements with which you may agree or disagree. Please indicate your level of agreement with each statement.

Versión original en español / Original version in Spanish

		Totalmente en Desacuerdo	Bastante en Desacuerdo	En Desacuerdo	Ni de acuerdo ni en desacuerdo	De Acuerdo	Bastante de Acuerdo	Totalmente de Acuerdo
1	Estoy dispuesto/a a realizar cualquier cosa para ser un/a emprendedor/a	☐	☐	☐	☐	☐	☐	☐
2	Mi objetivo profesional es convertirme en un/a emprendedor/a.	☐	☐	☐	☐	☐	☐	☐
3	Haré todo el esfuerzo posible para comenzar y llevar a cabo mi propia empresa	☐	☐	☐	☐	☐	☐	☐
4	Estoy decidido/a a crear una empresa en el futuro.	☐	☐	☐	☐	☐	☐	☐
5	Tengo pensado muy seriamente iniciar un negocio	☐	☐	☐	☐	☐	☐	☐
6	Tengo la firme intención de comenzar una empresa algún día.	☐	☐	☐	☐	☐	☐	☐

Versión en inglés / English version

		Strongly Disagree	Moderately Disagree	Disagree	Neither Agree nor Disagree	Agree	Moderately Agree	Strongly Agree
1	I am willing to do whatever it takes to become an entrepreneur.	☐	☐	☐	☐	☐	☐	☐
2	My professional goal is to become an entrepreneur.	☐	☐	☐	☐	☐	☐	☐
3	I will make every possible effort to start and run my own business.	☐	☐	☐	☐	☐	☐	☐
4	I am determined to start a business in the future.	☐	☐	☐	☐	☐	☐	☐
5	I have seriously considered starting a business.	☐	☐	☐	☐	☐	☐	☐
6	I firmly intend to start a business someday.	☐	☐	☐	☐	☐	☐	☐
